# Climate change knowledge, concerns and experiences in secondary school learners in South Africa

**DOI:** 10.4102/jamba.v14i1.1162

**Published:** 2022-06-10

**Authors:** Alison Kutywayo, Matthew Chersich, Nicolette P. Naidoo, Fiona Scorgie, Likho Bottoman, Saiqa Mullick

**Affiliations:** 1 Wits Reproductive Health and HIV Institute, Faculty of Health Sciences, University of the Witwatersrand, Johannesburg, South Africa; 2 Director Social Cohesion and Equity in Education, Department of Basic Education, Pretoria, South Africa

**Keywords:** climate change, education, GAP year, global warming, impact, knowledge, secondary schools, South Africa, youth

## Abstract

Climate change poses a major threat to the future of today’s youth. Globally, young people are at the forefront of climate change activism. Their ability to engage, however, depends on the level of knowledge of climate change and concern about the topic. We sought to examine levels of knowledge and concerns about climate change among youth in South Africa, and their experiences of heat exposure. Ten questions on climate change knowledge, concerns and experiences were nested within a cross-sectional survey conducted in a cluster randomised trial among 924 secondary school learners in 14 public schools in low-income Western Cape areas. Learners’ mean age was 15.8 years and they were predominately female. While 72.0% of respondents knew that climate change leads to higher temperatures, only 59.7% agreed that human activity is responsible for climate change, and 58.0% believed that climate change affects human health. Two thirds (68.7%) said that climate change is a serious issue and 65.9% indicated action is needed for prevention. Few learners indicated climate change events had affected them, although many reported difficulties concentrating during hot weather (72.9%). Female learners had lower knowledge levels than male learners, but more frequent heat-related symptoms. Learners scoring high on knowledge questions expressed the most concern about climate change and had the highest heat impacts. Many youth seem unaware that climate change threatens their future. Heat-related symptoms are common, likely undermining educational performance, especially as temperatures escalate. More is needed to mainstream climate change into South African school curricula.

## Introduction

Raising awareness of climate change and of the importance of protecting the environment are likely to be critical for mounting an effective response to climate change and for full engagement with mitigation and adaptation initiatives. Several studies in Africa suggest that familiarity with the term ‘climate change’ and general knowledge about climate change among adults and children are concerning, with negative implications for action (Lee et al. 2015; Nigatu, Asamoah & Kloos 2014). In a survey by Afrobarometer in 2018, for example, only 46% of South Africans had ever heard of the term climate change, with levels lowest among women (AfroBarometer 2019). In another survey of people aged 18–35 years, conducted by The Royal Institute of International Affairs in 2019 across 13 African countries, only slightly more than 10% felt that climate change was an important issue facing their country (Chatham House, The Royal Institute of International Affairs 2019). High rates of knowledge and concern have, however, been recorded among middle-class people in a study in Nigeria (Asiyanbi 2015) and in rural populations in Côte d’Ivoire (Yeo et al. 2016). Among university students in South Africa, levels of knowledge about climate change are also low (El Zoghbi & El Ansari 2014). Furthermore, only half the health faculty students in a study in Ethiopia could identify any specific health conditions linked to climate change (Nigatu et al. 2014).

Climate change represents a classic multidimensional global problem characterised by diverse actors, multiple stressors and complex time scales (Mudombi et al. 2017). Such challenges have been viewed as ‘wicked problems’, where there is incomplete information, and large differences between stakeholders regarding perceptions of the nature of the problem, the need for action and the type of action that should be taken (Stang & Ujvari 2015). Adding to this complexity is the fact that the trajectories of the different aspects of climate change, as well as of human behaviour, are unpredictable. Yet for different actors to work towards a common goal it is important that they have a shared understanding of the challenge or at least some converging perceptions on this (Mudombi et al. 2017). A common understanding of the climate crisis may also help to promote community or collective resilience (Toan do et al. 2014).

Solving the above complexities begins by building a solid foundation of knowledge around climate change among school learners. Building this knowledge is also a target of the Sustainable Development Goals, specifically target 13.3 – ‘improve education, awareness-raising and human and institutional capacity on climate change mitigation, adaptation, impact reduction and early warning’.

It is important to track progress towards these targets through, for example, evaluating levels of knowledge about climate change among youth, assessing whether youth are concerned about climate change and are experiencing impacts of climate extremes. Taken together, this information will help inform the planning of educational initiatives and health sector programmes to assist people to cope and adapt to a changing climate. We thus sought to measure levels of knowledge and concern about climate change through a quantitative survey among youth in South Africa, as well as what experiences they have had with climate change and extreme heat.

## Methods

### Study design and population

A cross-sectional survey, part of an endline evaluation of a trial, was conducted from July to October 2019 among female and male Grade 10 learners from 14 secondary schools in Khayelitsha, a peri-urban township about an hour’s drive from the Cape Town city centre. The schools are a sub-set of those enrolled in a cluster randomised controlled trial called the Girls Achieve Power Trial (GAP Year Trial) that began in April 2017 (Kutywayo et al. 2018; Wits Reproductive Health and HIV Institute 2021). In brief, the GAP Year trial tested the effectiveness of a comprehensive sexuality education, asset-building intervention in 26 public sector schools across three sites (Soweto and Tembisa in Gauteng province, and Khayelitsha in the Western Cape province). The aim was to reduce school dropout among adolescent girls between grades 8–10, while shifting gender attitudes and encouraging positive behaviour change among adolescent boys. These townships where the study was set are large, sprawling low to middle-income residential areas that were former black settlements under Apartheid. These settings were chosen based on high levels of HIV infections, gender-based violence and unintended pregnancy among learners in these areas. Schools were selected using the following inclusion criteria: mixed sex public high schools in Tembisa, Soweto and Khayelitsha; in quintiles 1–3 which had not been exposed to any asset building interventions in the past 6 months. Classes are overcrowded with a mean of 49 learners per class and more than one learner sharing a desk. The project placed a strong emphasis on stakeholder engagement to optimise participatory practices throughout the trial (Kutywayo et al. 2018). The GAP Year intervention did not include information on climate change or the environment.

All GAP Year participants, now in Grade 10 (2 years after the beginning of the trial) at the 14 selected schools in Khayelitsha were eligible to participate in this survey, irrespective of sex, age or race. Only those who had completed the baseline survey, were present on the day of data collection, and had parental consent and provided individual assent were recruited. Results for Soweto and Tembisa sites were not yet available at the time of writing; therefore, we only present the results for Khayelitsha learners.

### Study measures and overall scores

The survey was administered by a fieldworker who captured the responses directly onto a hand-held tablet. Questions were asked about participant socio-demographics, social support, gender norms, sexuality, perceptions of school safety and care seeking, as well as the climate change indicators. The survey was developed in English and later translated into IsiXhosa, the dominant South African language at the study sites. In this article we only report data on the population characteristics and responses to the climate change questions.

A quantitative approach was adopted: questions on climate change were presented as a series of nine statements to which respondents had to reply Agree, Neutral or Disagree. The statements covered three domains, namely, knowledge (four measures), concerns around climate change (two measures), and experiences of climate change and heat exposure (three measures). The indicators used to evaluate knowledge assessed whether the youth knew the causes of climate changes, and its manifestations in the natural world and in humans. Questions on concerns about climate change were formulated to evaluate the learner’s perceptions of the seriousness of phenomenon, and of the need for taking action. Finally, students were asked to report on whether climate change or extreme weather events influence their behaviour, and on how heat exposure affects their aggression levels and ability to concentrate in school.

### Data management and analysis

Survey data were collected and managed using REDCap (Research Electronic Data Capture) electronic data capture tools hosted at Wits Reproductive Health and HIV Institute (Wits RHI) (Harris et al. 2009, 2019). Survey data were stored on encrypted password-protected tablets and synced with secure servers at the Wits RHI research site. Data from REDCap were exported into Stata version 13.1 (Stata-Corp, LP, College Station, TX) for analysis.

We used descriptive analyses to examine the overall frequency of responses and identify differences in the answers by socio-demographic characteristics. We dichotomised the responses into agree versus neutral or disagree. A chi-square test was used to compare responses between different population sub-groups. We also assessed whether learners who correctly answered all four knowledge questions had higher levels of concern or had experienced climate-related phenomenon than the other learners. Furthermore, each correct knowledge answer was assigned a score of one point and a score of zero for neutral or disagree. The learners’ scores were compared between population sub-groups using the Student’s *t* test.

### Ethical considerations

The University of the Witwatersrand Human Research Ethics Community granted ethics approval (reference number: M160940) and the Western Cape Department of Education and Gauteng Department of Education granted approval for the research activities.

## Results

A total of 924 learners participated in the survey. The mean number of participants per school was 66, ranging from 36 to 104. The mean age of the learners was 15.8 years (standard deviation = 0.9 years), with 21.8% of learners aged 17 years and older (201/922). Most learners were female (67.7%; 626/924) and IsiXhosa speaking (89.7%; 828/923).

Of the 924 learners, only 39.6% lived with both parents (366), 42.6% lived with one of their parents (394) and 17.7% stayed with someone other than a parent (164). About a quarter reported that their parents or guardian were unemployed (244/924; 26.4%).

### Climate change knowledge, concerns and experiences

Although 72.0% of respondents stated that climate change leads to higher temperatures, only 59.7% agreed that human activity is a cause of climate change (Table 1). Furthermore, 80.7% knew that climate change can affect people, plants and animals, but only 58.0% believed that climate change affects human health. In total, 37.1% answered all four knowledge questions correctly (341/919).

**TABLE 1:** Knowledge, concerns and experiences of climate change among secondary school learners in the Western Cape, South Africa.

Indicator	All learners % agree	*n*	Females % agree	*n*	Males % agree	*n*
**Knowledge about climate change**						
Climate change causes temperatures to rise	72.0	666	70.9	444	74.5	222
Human activity is a cause of climate change	59.7	552	57.6†	361	64.1†	191
Climate change affects people, plants and animals	80.7	743	79.8	499	82.7	244
Climate change affects my health	58.0	536	57.8	362	58.5	174
**Levels of concern about climate change**						
Climate change is a serious issue	68.7	635	68.0	426	70.1	209
Action should be taken to prevent climate change	65.9	609	64.2	402	69.4	207
**Experiences of climate change and heat exposure**						
Climate change events (e.g. heat and drought) influence my behaviour	38.5	356	38.9	244	37.5	112
It is hard to concentrate in school when it is too hot	72.9	674	76.6*	480	65.1	194
When it is hot, I am more likely to scream, push or punch others	13.9	128	14.2	89	13.1	39

Note:

* , *p* < 0.05.

† , *p* is 0.05 to 0.1.

Only slightly more than two thirds said that climate change is a serious issue (68.7%), and a similar figure held the view that action is needed to prevent climate change (65.9%). Of those who believed that climate change was a serious issue, 82.2% felt that action should be taken (522/609). Overall, 21.9% felt that climate change was not a serious issue, and that action was not needed (202/924).

Less than half the learners reported that climate change events such as heat exposure or droughts had influenced or impacted on their behaviour (38.5%). However, almost three quarters of respondents said that they had difficulties concentrating during periods of high temperatures (72.9%). There was considerable variation across schools in this measure, with the proportion of participants reporting concentration difficulties ranging from 60.0% in one of the schools (30/50) to a high of 90.5% in another (38/42). A minority of learners reported links between heat exposure, and aggression or physical violence (13.9%), although levels reached 18–19% in three schools.

**FIGURE 1:**
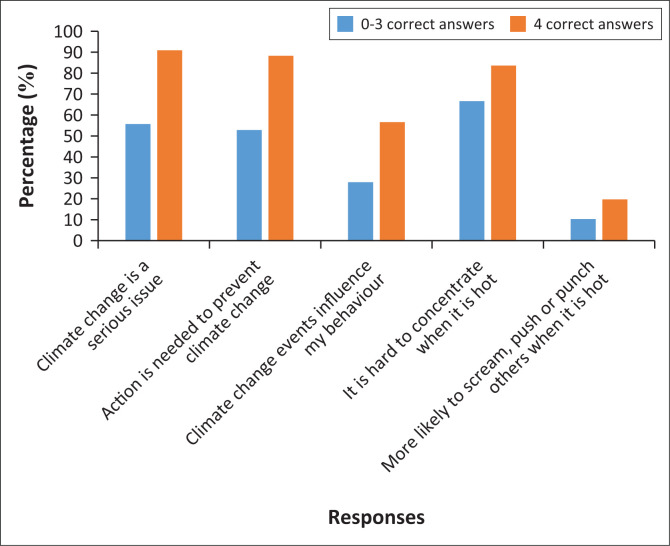
Comparison of knowledge levels and climate-related experiences between pupils with high and low levels of knowledge about climate change.

Links between knowledge, concerns and climate-related experiences were detected at school and individual level. Learners at the school with the lowest knowledge scores also were least concerned about climate change and the converse was true in the school with the highest knowledge levels. Participants who correctly answered all the knowledge questions were more concerned about climate change and also had the highest reported climate-related experiences (Figure 1).

### Factors associated with climate change knowledge, concern about climate change and climate-related experiences

Few associations were detected between the study outcomes and socio-demographic factors, such as age, being born in the province and parent employment status. There were two statistically significant sex differences. Female learners performed worse than male learners on knowledge indicators, with female learners having a mean 0.20 lower knowledge score than male learners (*p* = 0.04). Female participants were, however, more likely to report difficulties about concentrating during hot weather: about three quarters of female participants (76.6%) experienced these difficulties compared to only 65.1% of male participants (*p* < 0.001).

We did not detect any associations between age and levels of knowledge or concern about climate change. Participants below 16 years, however, reported the larger impacts of high temperatures on concentration (76.5%, 295/673) than older participants (70.4%, 378/537; *p* = 0.04).

## Discussion

Our study found major gaps in knowledge about the causes and manifestations of climate change among Grade 10 learners in low-income communities in the Western Cape. Changes in climate were commonly not ascribed to human activity, nor necessarily viewed as requiring major shifts in societal or individual actions. The quantitative survey findings indicate that a considerable portion of learners appeared to be unconcerned about climate change, with only two thirds regarding it as a serious issue. One fifth believed both that climate change was not a serious issue and that no preventative action was needed. Encouragingly however, those who demonstrated high knowledge of climate change saw the urgency of climate change action. Strong links between knowledge and levels of concern and desire for action, which we found at both individual and school level, suggests that school learning or public information campaigns that raise knowledge may result in attitudinal shifts, or that having personal experiences of climate change may generate an increased interest in the topic (Toan do et al. 2014). We strongly suggest that education campaigns are needed, ensuring that they provide appropriate awareness in the local language.

Several studies in Africa indicate that a large portion of the population had observed changes in temperature and rainfall patterns over the past decades (Ajuang et al. 2016; Balama et al. 2016; Dah-Gbeto & Villamor 2016; Debela et al. 2015; Habtemariam et al. 2016; Joshua et al. 2016; Kupika et al. 2019; Mayala et al. 2015; Mertz et al. 2012; Mulenga, Wineman & Sitko 2017; Ncube & Tawodzera 2019; Oruonye 2011) although less so among the youth (Debela et al. 2015). There is much less certainty, however, about whether human activity is responsible for these changes. In our survey, similar to a recent study in Zimbabwe (Ncube & Tawodzera 2019), 40% of respondents did not know that climate change was due to human activity. In another study in Zimbabwe by Kupika et al, people in rural areas attributed climate change to ‘sin’, ‘a mystical phenomenon’, ‘spiritual forces’ or to ‘negative’ cultural changes that have occurred in recent times (Kupika et al. 2019). Moreover, knowledge that the climate is changing does not necessarily translate into a conviction that actions are required (Limantol et al. 2016). In an Afrobarometer survey among adults across South Africa, only 52% of those familiar with the term ‘climate change’ believed it needed to be stopped (AfroBarometer 2019).

Many learners, in particular female and younger learners, drew clear links between difficulties in concentrating and heat exposure, and some noted heat-aggression associations. Making connections between rising global temperatures and symptoms of heat stress could potentially assist learners to comprehend the importance and urgency of addressing climate change. In many schools in South Africa, classrooms are made from converted shipping containers or prefabricated sheeting with corrugated iron roofs (Bidassey-Manilal et al. 2016). Often with poor insulation and little natural ventilation, these classrooms can have as many as 50 learners in a class, who themselves generate a considerable heat load (Ellis et al. 1978). In one study in Johannesburg, which has a relatively mild climate, temperatures reached as high as 47.5°C in the containers and many students reported experiencing heat-health symptoms, including drowsiness, poor concentration, and thirst (Bidassey-Manilal et al. 2016). The impacts of high temperatures on educational performance are very concerning. In a meta-analysis involving 18 studies, the authors calculated that students in classrooms with an indoor temperature of 30°C scored 20% lower on tests than those in classes around 20°C (Epstein et al. 1980). Teachers exposed to high temperatures may also become lethargic and irritable (International Labour Organization 2019). Studies in high-income countries have also shown that the effects of heat on education performance can be ameliorated by air conditioning (Schnippel et al. 2015) and mechanical ventilation (Porras-Salazar et al. 2018). Further research is needed to identify effective interventions other than air conditioning. These could potentially include providing cold water at regular prespecified intervals, improved natural ventilation, substituting closed shoes with sandals, using light-coloured, loose clothing, and adapting school hours during the hottest seasons, with either earlier starting times or a longer lunchtime break and later finishing hours (Chersich et al. 2019). Furthermore, planting trees within the school grounds provides shade and cooling, and other health and environmental benefits. A ‘cooling room’ could be developed in the school, with fans installed on the walls, the roofs painted white, and trees planted nearby. During extremely hot days learners could spend time in the cooling room during breaks or after school, and the room could be used for examinations. Interventions such as these may assist learners to draw links between extreme heat and climate change, and perhaps heighten their awareness of the need for action.

Youth have major stressors in South Africa, including violence and mental health conditions, which may be worsened by climate change (Cheng et al. 2014; Scorgie et al. 2017). Extreme heat and its impacts on aggression may further augment violence in schools as noted by some learners and in a large body of literature (Chersich et al. 2019) as well as from other data from the GAP Year trial. In the learners participating in this survey, for example, as many as 26% had ever experienced violence, with physical violence perpetrated most commonly by peers at school (analysis of GAP Year data, presented in Kutywayo et al. (2021).

Clearly more is needed within secondary schools to address gaps in awareness around climate change. The school curriculum in South Africa currently includes only very basic information about the topic, and makes no mention of the human health impacts of this phenomenon (Department of Basic Education Republic of South Africa 2011). Comprehensive integrated learning approaches which also involve the community such as the Care and Support for Teaching and Learning framework, have shown particular promise in environmental education (MIET AFRICA 2018). Encouragingly, the Western Cape Education Department has recently launched an e-book on climate change in three local languages, providing another platform to advance a climate-resilient future for the sector (Department of Agriculture Western Cape province South Africa 2021). Raising ‘climate literacy’ among learners may also help to improve the overall understanding of climate change within the broader community, as youth are an important vehicle for knowledge transfer across generations. Curriculum reform is also needed at tertiary level institutions. Of particular concern, limited attention has been given to the topic of climate change in the training of health workers (Cruz et al. 2018; Nigatu, Asamoah & Kloos 2014) and levels of knowledge among health science students, health workers and even environmental health practitioners is low in some settings in Africa more broadly (Abaya, Mandere & Winqvist 2011; Cruz et al. 2018; Felicilda-Reynaldo et al. 2018; Nigatu et al. 2014; Shezi et al. 2019).

Heightened knowledge about climate change may stimulate activism among South African youth, much like the activism that has had powerful impacts in many other parts of the world. Increased awareness and community engagement could help shape decision-making at local and national level and may increase the uptake of adaptation and mitigation strategies within communities. Where policymakers perceive low awareness or understanding of climate change among local communities, they may be more likely to overlook or even actively exclude communities in the co-design and then co-delivery of adaptation projects (Mudombi et al. 2017).

However, generating awareness about climate change, raising levels of concern, and developing a common understanding of the problem is not straightforward. The complex terminology used in describing climate change may not be accessible to many school learners. This is a challenging topic for learners as a range of complex climate change narratives are being presented by multiple actors, including the fossil fuel industry (Creamer Media’s Mining Weekly 2021; Hathaway & Maibach 2018; Hussey & Arku 2019). The school curriculum in South Africa does little to address these complexities (Department of Basic Education Republic of South Africa 2011). A previous study in South Africa suggested that information campaigns should focus on communicating solutions and strategies, rather than highlighting the risks faced, and on providing an understanding of what climate change ‘really means’, even though the complexity of the topic makes this difficult to break the topic into a few key messages (El Zoghbi & El Ansari 2014). It may thus be important to tailor teaching around climate change to specific settings and focus on highlighting local impacts on people’s health and future quality of life. High levels of social and political mistrust, and the framing of climate change as a ‘Western or white’ notion pose challenges to communication initiatives (El Zoghbi & El Ansari 2014). Additionally, short-term, more immediate priorities such as poverty, school violence and coronavirus disease 2019 (COVID-19) make it understandable that longer term concerns such as climate change are perceived as less pressing (Chatham House, The Royal Institute of International Affairs 2019).

The strengths of the study include a large sample from several schools, set within a project that has much experience in securing high-quality data from study participants and strong relations with the learners and teachers built up over several years. The experiences of learners in the Western Cape, however, may not reflect those of learners elsewhere given the recent drought experiences and relatively better functioning of schools in the province. Knowledge levels may also be higher in quintile 4 and 5 schools who were excluded from the study. Other limitations include the fact that the validity of the measures used in this study is uncertain. Measures of knowledge and experiences about climate change have not been validated, and thus the content and construct validity of our measures are not known. Researchers and international agencies need to select a standardised, validated set of indicators of knowledge and perceptions which can be applied in future. Lastly, although close links between knowledge and concerns suggest that advancing knowledge may have ripple effects, that conclusion may be over-simplistic as for many people there is a disconnect between knowledge, attitudes, and actions.

## Conclusion

Clearly much more needs to be done to educate learners in South Africa about climate change and its consequences, including through urgent reform of school curriculum. South Africa is not on track to reaching the Sustainable Development Goals on climate change education as well as education commitments made in the Lima Declaration on Education and Awareness on Climate Change, and Paris Agreement (United Nations Framework Convention on Climate Change 2014). More than a quarter of learners lacked basic knowledge of the topic and its links with raised temperatures, and even fewer understood the anthropogenic nature of the phenomenon. Scientific, cultural and social considerations are important when designing teaching curricula and educational tools. The robust linkages between knowledge and desire for action on climate change noted in this study provides optimism that raising knowledge levels may influence concerns and understandings of climate change.
